# Three members of *Opisthomonorcheides* Parukhin, 1966 (Digenea: Monorchiidae) from carangid fishes (Perciformes) from Indonesia, with a review of the genus

**DOI:** 10.1007/s11230-017-9717-5

**Published:** 2017-03-23

**Authors:** Rodney A. Bray, Harry W. Palm, Scott C. Cutmore, Thomas H. Cribb

**Affiliations:** 1 Department of Life Sciences, Natural History Museum, Cromwell Road, London, SW7 5BD UK; 2 Faculty of Agricultural and Environmental Sciences, Aquaculture and Sea-Ranching, University of Rostock, Justus-von-Liebig-Weg 6, 18059 Rostock, Germany; 3 Faculty of Veterinary Sciences, Udayana University, Bukit Jimbaran, 80363 Badung, Bali Indonesia; 4 School of Biological Sciences, The University of Queensland, St Lucia, QLD 4072 Australia

## Abstract

Three species of *Opisthomonorcheides* Parukhin, 1966 are reported for the first time from Indonesian waters: *O. pampi* (Wang, 1982) Liu, Peng, Gao, Fu, Wu, Lu, Gao & Xiao, 2010 and *O. ovacutus* (Mamaev, 1970) Machida, 2011 from *Parastromateus niger* (Bloch), and *O. decapteri* Parukhin, 1966 from *Atule mate* (Cuvier). Both *O. pampi* and *O. ovacutus* can now be considered widespread in the Indo-Pacific region, with earlier records of these species being from Fujian Province, China and Penang, Malaysia, respectively. We redescribe *O. decapteri* from one of its original hosts, *Atule mate*, off New Caledonia, and report this species from Jakarta Bay, Indonesia, extending its range throughout the Indian Ocean into the south-western Pacific. All three species possess a genital atrium that is long, sometimes very long, and a genital pore that is located in the forebody. This validates the interpretation that the original description was erroneous in reporting the genital pore in the hindbody, well posterior to the ventral sucker. These observations verify the synonymy of *Retractomonorchis* Madhavi, 1977 with *Opisthomonorcheides*. A major discrepancy between the species of *Opisthomonorcheides* is that some are described with the uterus entering the terminal organ laterally and some with it entering terminally; this feature needs further analysis. Based on the length of the genital atrium and the posterior extent of the vitellarium, the 27 species of *Opisthomonorcheides* considered valid can be divided into four groups. Among the 53 host records analysed, the families Carangidae (53% of records), Stromateidae (17%) and Serranidae (5.7%) are the most common; the reports are overwhelmingly from members of the Perciformes (91%), with further records in the Clupeiformes (5.7%), Gadiformes (1.9%) and Pleuronectiformes (1.9%). Two fish genera (*Parastromateus* Bleeker and *Pampus* Bonaparte) dominate the recorded hosts, with the black pomfret *Parastromateus niger* harbouring six species, the silver pomfret *Pampus argenteus* (Euphrasen) harbouring six, and the Chinese silver pomfret *P. chinensis* (Euphrasen) two. A host-parasite checklist is presented. We discuss the host-specificity of members of the genus, questioning some records such as that of *O. decapteri* in a deep-sea macrourid. We also comment on the morphological similarity, but phylogenetic distance, between the various Pomfret species, advancing the possibility that a series of host misidentifications has occurred. Sequences of the ITS2 rDNA gene generated for *O. pampi* and *O. ovacutus* are briefly discussed and molecular data are lodged in the GenBank database.

## Introduction

Fish parasitological studies in Indonesian waters demonstrate the difficulty of an enormous biodiversity confronted by a meagre research effort and a lack of competent investigations. Although several new parasite species have been described in recent years (e.g. Palm, 2004; Bray & Palm, 2009; Yong et al., 2016), the fauna remains largely unknown (Cribb et al., 2016); it can be expected that many more species await description and many other known species from the Indo-Pacific region also occur off Indonesia (see Kuchta et al., 2009). Consequently, taxonomic treatments of nearly all parasite taxa in the region are needed.

During the First Educational Workshop on Fish Parasitology in Indonesia during 2013, Indonesian and international students and researchers investigated a wide range of fishes from Balinese waters. We herein report three species of *Opisthomonorcheides* Parukhin, 1966, a genus not yet recorded off Indonesia, from two carangid species. This genus was erected by Parukhin (1966) for a species reported from *Decapterus* sp. and *Atule mate* (Cuvier) in the Gulf of Tonkin, described with a genital pore in the hindbody well posterior to the ventral sucker. Madhavi (1977) later described a new genus, *Retractomonorchis* Madhavi, 1977 (for *R*. *delicatus* Madhavi, 1977), from *Pampus chinensis* (Euphrasen) and *P. argenteus* (Euphrasen) off the Waltair Coast, India. She described the genus with a genital pore in the posterior forebody and a thin-walled genital atrium reaching into the hindbody. In some cases, the genital atrium was difficult to detect and she indicated that, if it had been missed by Parukhin (1966), then her worms were congeneric with his. Hafeezullah (1984) studied over 40 specimens of *Opisthomonorcheides karwarensis* Karyakarte & Yadav, 1976 and also found the genital atrium difficult to distinguish in some cases and, presuming Parukhin (1966) to be mistaken, synonymised *Retractomonorchis* with *Opisthomonorcheides*. In her review of the Monorchiiidae, Madhavi (2008) agreed with Hafeezullah’s conclusions and also synonymised *Longimonorchis* Mamaev, 1970 with *Opisthomonorcheides*. She also considered *Tritesticulum* Bilqees, 1971 a synonym, but says that ‘its description is inadequate and probably erroneous’. Taking Bilqees’ (1971) description at face value, it does not resemble species of *Opisthomonorcheides*, but the worm is probably unrecognisable. Species of *Opisthomonorcheides* are clearly distinguished from species of the two other genera of the subfamily Opisthomonorcheidine Yamaguti, 1971 by the presence of a single testis rather than two testes (Madhavi, 2008).

Machida (2011) described worms identified as belonging to the type-species of *Opisthomonorcheides*, *O. decapteri* Parukhin, 1966, from *Rastrelliger kanagurta* (Cuvier) off Palawan, the Philippines, with a long genital atrium and the genital pore in the posterior forebody. We have available some specimens which we consider *O. decapteri*, from one of its original hosts, *Atule mate* (no type-host was designated) originating from the Fish Market in Nouméa, New Caledonia, and Jakarta Bay, Indonesia. We include a description and illustration of these worms to show that the genital pore is in the forebody and the genital atrium is long, thus validating the presumptions of Madhavi (1977, 2008) and Hafeezullah (1984) and agreeing with Machida (2011). The species *O. pampi* (Wang, 1982) Liu, Peng, Gao, Fu, Wu, Lu, Gao & Xiao, 2010, *O. ovacutus* (Mamaev, 1970) Machida, 2011 and *O. decapteri* are described for the first time from Indonesian waters.

## Materials and methods

During the First Educational Workshop on Fish Parasitology in Indonesia, July 21st - August 2nd, 2013, at the Faculty of Veterinary Sciences, Denpasar, Bali, Indonesia, a single fresh specimen of *Parastromateus niger* (Bloch) was bought at Kedonganan fish market and transported on ice to the laboratory for study. Digeneans were collected live, immediately fixed in nearly boiling saline and stored in 70% ethanol. Whole-mounts were stained with Mayer’s paracarmine, cleared in beechwood creosote and mounted in Canada balsam. Specimens of *Atule mate* were collected from Jakarta Bay on the 12th of January, 2012, deep frozen and transported to UNSOED University, Purwokerto, for subsequent study. A specimen of *P. niger*, originating from off Tegal, northern coast of Central Java, was collected in June, 2011 at the fish market in Cilacap, south coast of Central Java, and studied in the laboratory of UNSOED University, Purwokerto. Whole-mounts of trematodes from these hosts were fixed and stored in 70% ethanol and later mounted in glycerine.

Measurements were made using a drawing tube attached to an Olympus BH-2 microscope, using a Digicad Plus digitising tablet and Carl Zeiss KS100 software adapted by Imaging Associates, and are given in micrometres. Measurements taken from worms from frozen hosts are not included in the tables. The following abbreviations are used: BMNH, the British Museum (Natural History) Collection at the Natural History Museum, London, UK; NBC, National Biodiversity Collection, Lipi-Cibinon, Bogor, Java, Indonesia; ZMB, Natural History Museum, Berlin, Germany; MNHN JNC, Muséum National d’Histoire Naturelle, Paris, France.

Genomic DNA of two species, *O. pampi* and *O. ovacutus*, was isolated and purified from individual specimens using a genomic DNA extraction kit (Peqlab Biotechnology GmbH, Erlangen, Germany) according to the manufacturer’s instructions. The ITS2 rDNA region was amplified by using the forward primer 3S (5′-GGT ACC GGT GGA TCA CGT GGC TAG TG-3′) (Morgan & Blair, 1995) and the reverse primer ITS2.2 (5′-CCT GGT TAG TTT CTT TTC CTC CGC-3′) (Cribb et al., 1998). PCR-reactions (25 μl) included 2 μl of genomic DNA and 23 μl of Master-Mix (Peqlab Biotechnology GmbH, Erlangen, Germany) containing 4 mM MgCl_2_, 40 mM Tris-HCl (pH 8.55), 32 mM (NH_4_)_2_SO_4_, 0.02% Tween 20 (ready mixed as Peqlab “Reactionbuffer Y”, 2.5 µl) and 2.5 u Taq-Polymerase (0.5 µl), 0.4 mM dNTP (1.25 µl) and 16.75 µl water and 1 μl of each primer (10 pmol/µl). Amplification was performed in a thermocycler (Biozym Scientific GmbH, Germany) under the following conditions: an initial single cycle of 95°C denaturation for 3 min, 45°C annealing for 2 min, 72°C extension for 90 s, followed by 4 cycles of 95°C denaturation for 45 s, 50°C annealing for 45 s, 72°C extension for 90 s, followed by 30 cycles of 95°C denaturation for 20 s, 52°C annealing for 20 s, 72°C extension for 90 s, followed by a final 72°C extension for 5 min (Cutmore et al., 2013). Control samples without DNA were included in each PCR run. PCR products were examined on 1% agarose gels. A 100 bp ladder marker (Peqlab Biotechnology GmbH, Erlangen, Germany) was used to estimate the size of the PCR products. To identify the specimens, the PCR products were purified with Cycle-Pure Kit (Peqlab Biotechnology GmbH, Erlangen, Germany) and sequenced by Seqlab (Goettingen GmbH, Germany) using 3 μl of primer (of each 3S and ITS2.2) and 7 μl of the PCR product (~ 250 ng/μl). Sequencher™ version 4.5 (GeneCodes Corp.) was used to assemble and edit contiguous sequences and the start and the end of the ITS2 region were determined by annotation through the ITS2 Database (Keller et al., 2009; Ankenbrand et al., 2015) using the ‘Metazoa’ model. Sequences were deposited in the GenBank database under the accession numbers KX839157 (*O. ovacutus*) and KX839158 (*O. pampi*).

**Family Monorchiidae Odhner, 1911**

**Genus** ***Opisthomonorcheides*** **Parukhin,** **1966** 1

Syns *Longimonorchis* Mamaev, 1970^2^; (?) *Tritesticulum* Bilqees, 1971^3^; *Retractomonorchis* Madhavi, 1977^4^

***Opisthomonorcheides pampi*** **(Wang,** **1982** **) Liu, Peng, Gao, Fu, Wu, Lu, Gao & Xiao, 2010**

Syn. *Longimonorchis pampi* Wang, 1982^5^

*Host*: *Parastromateus niger* (Bloch) (Carangidae).

*Site in host*: Intestine.

*Localities*: Off Bali (Bali Fish Market) (29.vii.2013); off Cilacap, Java (7°43′41″S, 109°0′21″E; from frozen host).

*Voucher specimens*: NBC MZBTRr 238-239, ZMB E.7612-7614, BMNH 2016.10.7.2.

*Representative DNA sequences*: ITS2 rDNA, two identical replicates (one submitted to GenBank KX839158).

*Type-host and locality*: *Pampus argenteus* (Euphrasen) (Stromateidae) (first host listed), off Fujian Province, China.

*References*: 1. Wang (1982); 2. Present study.

*Hosts*: Stromateidae: *Pampus argenteus* (Euphrasen) (1); Carangidae: *Parastromateus niger* (Bloch) (2); Percichthyidae: *Lateolabrax japonicus* (Cuvier) (1).

*Distribution*: South China Sea off Fujian Province, China (1); Off Bali (2); Indian Ocean off Java (2).

### Description (Figs. 1–2)

[Based on 5 specimens; measurements of 4 specimens from Bali Fish Market presented in Table 1.] Body narrow, elongate, widest in region of gonads. Body spines tiny, in annular rows, reaching to about cirrus-sac region. Oral sucker subglobular, subterminal, small. Ventral sucker rounded, larger than oral sucker. Forebody short. Prepharynx long, narrow. Pharynx squat-pyriform, small. Oesophagus short, wide. Intestinal bifurcation in posterior forebody. Caeca terminate close to posterior extremity, sometimes obscured by eggs.

**Fig. 1–5:**
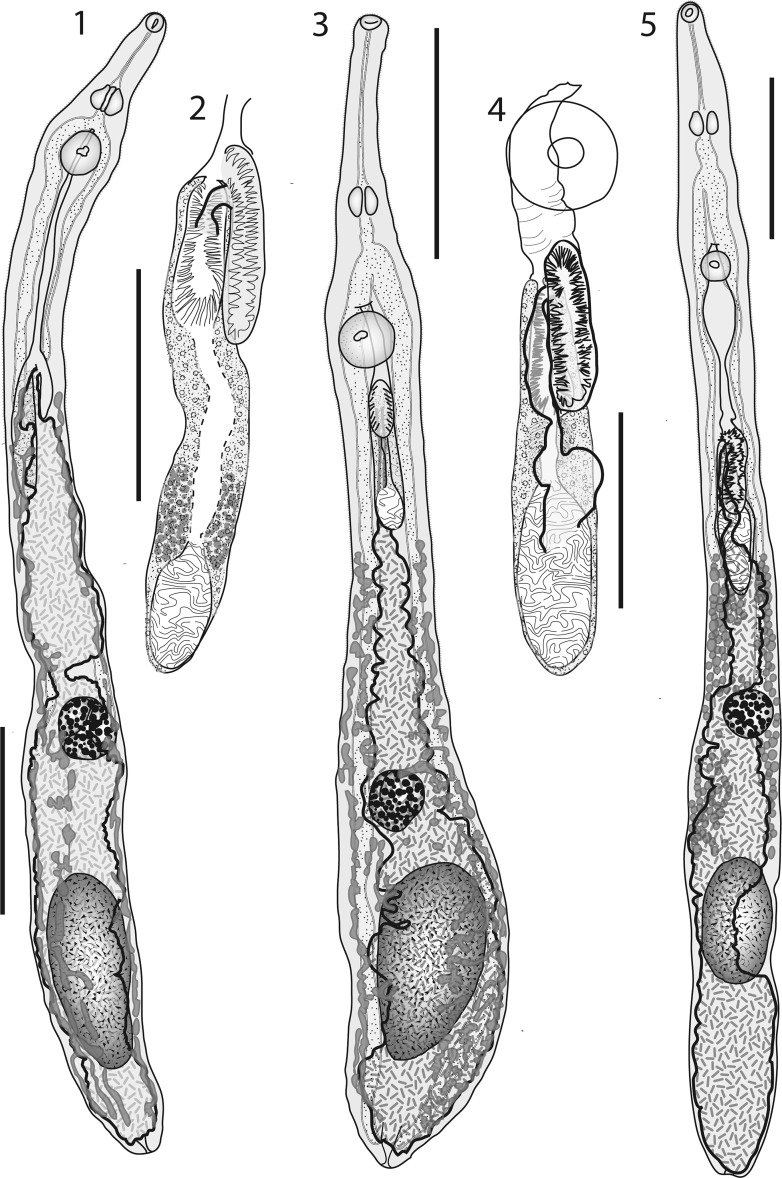
Line drawings of *Opisthomonorcheides* spp. collected in this study. 1, 2, *O. pampi* (Wang, 1982), ventral view, uterus in outline (1), terminal genitalia (2). 3, 4, *O. ovacutus* (Mamaev, 1970), ventral view, uterus in outline (3), terminal genitalia (4). 5, *O. decapteri* Parukhin, 1966, ventral view, uterus in outline. *Scale-bars*: 1, 3, 5, 500 μm; 2, 4, 200 μm

**Table 1:** Measurements of *Opisthomonorcheides* spp.

Species	*O. pampi* (Wang, 1982)	*O. ovacutus* (Mamaev, 1970)	*O. ovacutus*	*O. decapteri* Parukhin, 1966
Host	*Parastromateus niger*	*Parastromateus niger*	*Parastromateus niger*	*Atule mate*
Locality	Off Bali	Off Bali	Off Penang, Malaysia	Off New Caledonia
	(n = 4)	(n = 6)	(n = 2)	(n = 3)
Body length	2,574–3446 (3,130)	2,037–2,893 (2,339)	1,551–3,192	3,128–3,630 (3,457)
Body width	253–391 (303)	224–383 (287)	226–406	269–281 (274)
Forebody length	317–402 (371)	494–654 (562)	365–692	692–789 (748)
Oral sucker	49–58 × 50–64 (51 × 56)	32–43 × 49–55 (39 × 52)	32–39 × 33–41	23–58 × 34–63 (45 × 52)
Prepharynx length	135–211 (178)	226–336 (266)	175–330	201–292 (254)
Pharynx	77–86 × 76–87 (82 × 82)	60–78 × 57–70 (69 × 64)	66–98 × 42–86	72–87 × 86–99 (79 × 92)
Oesophagus length	25–44 (32)	23–86 (56)	40–54	93–113 (102)
Intestinal bifurcation to ventral sucker	14–40 (31)	82–188 (131)	51–180	240–252 (247)
Pre-vitelline distance	835–1,203 (1,052)	826–1,137 (964)	658–1,338	1,665–1,777 (1,717)
Vitelline field length	1,587–2,130 (1,948)	943–1,649 (1,271)	772–1,549	848–1,033 (941)
Ventral sucker	123–140 × 100–127 (129 × 113)	100–129 × 101–127 (115 × 110)	98–138 × 98–151	98–105 × 98–110 (102 × 104)
Cirrus-sac	435–604 × 73–94 (501 × 83)	323–831 × 54–134 (460 × 77)	285–450 × 49–77	493–605 × 131–160 (544 × 148)
Ventral sucker to ovary	1,141–1,595 (1,407)	625–982 (775)	533–1,178	1,222–1,436 (1,314)
Ovary	148–154 × 116–239 (151 × 170)	102–161 × 115–164 (122 × 132)	95–186	128–144 × 130–166 (137 × 149)
Ovary to testis distance	217–320 (269)	57–149 (105)	74–75	285–483 (344)
Testis	462–595 × 153–248 (512 × 212)	314–477 × 181–253 (405 × 218)	214–566 × 153–322	251–383 × 169–223 (331 × 194)
Post-testicular distance	218–306 (261)	139–414 (250)	173–373	370–602 (469)
Post-vitelline distance	71–149 (103)	73–170 (111)	115–201	639–959 (815)
Post-uterine distance	11–42 (23)	21–74 (39)	29–34	14–60 (38)
Post-caecal distance	33–210 (121)	27–44 (35)	?–11	15–21 (18)
Eggs	19–26 × 11–15 (21 × 13)	18–21 × 12–14 (20 × 13)	16–18 × 10–11	16–18 × 9–11 (17 × 10)
Width (%)^a^	7.64–11.3 (9.73)	9.61–15.3 (12.3)	12.7–14.6	7.45–8.72 (7.97)
Forebody (%)^a^	11.4–12.3 (11.9)	21.9–25.9 (24.1)	21.7–23.6	21.0–22.1 (21.7)
Sucker length ratio	1:2.13–2.85 (2.54)	1:2.51–3.71 (3.00)	1:2.53–4.35	1:1.70–4.50 (2.69)
Sucker width ratio	1:1.56–2.40 (2.06)	1:1.96–2.30 (2.10)	1:2.39–4.58	1:1.66–3.25 (2.20)
Pharynx: oral sucker width ratio	1:1.19–1.61 (1.49)	1:1.13–1.28 (1.23)	1:1.01–2.61	1:1.56–2.55 (1.89)
Oral sucker length (%)^a^	1.42–191 (1.65)	1.38–2.00 (1.67)	0.99–2.50	0.75–1.60 (1.29)
Pharynx length (%)^a^	2.34–.18 (2.64)	2.54–3.49 (2.98)	3.08–4.24	1.97–2.47 (2.28)
Ventral sucker length (%)^a^	3.84–4.77 (4.16)	4.14–5.60 (4.97)	4.31–6.34	2.71–3.36 (2.97)
Oesophagus length (%)^a^	0.75–1.29 (1.02)	1.04–3.04 (2.37)	1.69–2.55	2.58–3.13 (2.94)
Pre-vitelline distance (%)^a^	32.4–34.9 (33.5)	37.5–46.5 (41.4)	41.9–42.4	47.1–53.2 (49.8)
Vitelline field length (%)^a^	60.6–64.5 (62.2)	46.3–5.70 (54.1)	48.5–49.8	26.0–28.6 (27.2)
Ovary length (%)^a^	4.37–5.76 (4.89)	4.40–7.00 (5.26)	5.81–6.11	3.55–4.61 (4.00)
Ovary to testis distance (%)^a^	7.01–9.68 (8.60)	2.60–5.93 (4.49)	2.33–4.86	9.10–10.5 (9.90)
Testis length (%)^a^	14.6–17.9 (16.4)	15.3–20.8 (17.4)	13.8–17.7	8.01–10.6 (9.49)
Post-testicular distance (%)^a^	7.75–9.27 (8.34)	6.08–14.3 (10.6)	11.1–11.7	11.8–16.6 (13.5)
Post-vitelline distance (%)^a^	2.15–4.32 (3.30)	2.91–8.36 (4.89)	6.30–7.42	20.4–26.4 (23.4)
Post-uterine distance (%)^a^	0.31–1.33 (0.75)	0.85–3.65 (1.73)	0.90–2.22	0.39–1.91 (1.13)
Post-caecal distance (%)^b^	7.46–83.6 (45.5)	10.0–23.6 (15.4)	?–2.91	4.06–4.87 (4.47)
Post-caecal distance (%)^a^	0.63–6.57 (3.60)	1.18–1.73 (1.44)	?–0.34	0.48–0.59 (0.53)
Cirrus-sac length (%)^a^	13.6–17.5 (15.1)	14.8–28.7 (18.1)	14.1–18.4	13.6–17.0 (15.8)
Ventral sucker to ovary (%)^a^	43.8–46.3 (44.9)	30.5–35.2 (33.1)	34.4–36.9	35.3–39.7 (38.0)
Intestinal bifurcation to ventral sucker (%)^c^	4.45–9.85 (8.12)	14.8–28.8 (23.3)	13.9–26.0	30.5–36.5 (33.2)
Prepharynx (%)^a^	5.24–6.40 (5.65)	8.35–13.4 (11.5)	10.3–11.3	6.41–8.09 (7.32)
Pre-bifurcal distance	303–367 (340)	368–503 (431)	314–512	440–548 (501)
Pre-bifurcal distance (%)^a^	10.3–11.8 (10.9)	16.1–20.5 (18.5)	16.0–20.3	14.1–15.2 (14.5)
Pre-ovarian distance	1,580–2,137 (1,907)	1,270–1,756 (1,452)	997–2,008	2,020–2,327 (2,164)
Pre-ovarian distance (%)^a^	59.9–62.0 (60.9)	59.6–65.3 (62.2)	62.9–64.3	59.1–64.6 (62.7)
Genital atrium length	400–714 (588)	112–204 (166)	49–152	534–605 (566)
Extent of genital atrium in hindbody	261–575 (453)	0–71 (37)	0	411–441 (431)
Terminal organ length	167–199 × 39–57 (181 × 47)	84–139 × 33–63 (108 × 45)	72–114 × 35–46	246–278 × 76–90 (266 × 84)
Genital atrium length (%)^a^	15.5–20.7 (18.6)	4.98–9.05 (7.24)	3.17–4.76	14.8–17.8 (16.4)
Extent of genital atrium in hindbody^d^	22.9–36.0 (31.6)	0–9.96 (5.11)	0	28.6–36.1 (33.0)

^a^percent of body-length; ^b^ percent of post-testicular distance; ^c^ percent of forebody length; ^d^ percent of ventral sucker to ovary distance

Testis single, oval, close to posterior extremity of body. Cirrus-sac large, claviform, reaching to about mid-point between ventral sucker and ovary. Internal seminal vesicle oval, in posterior quarter of cirrus-sac. Pars prostatica long, narrow, with distinct gland-cell ducts arcing around it. Ejaculatory duct wide, rectilinear, lined with long spines. Genital atrium long, narrow, reaches well into hindbody. Genital pore median, in posterior forebody.

Ovary clearly tripartite, although overall outline may be weakly tri-lobed or almost circular, in posterior half of hindbody, pre-testicular, distant from testis. Uterine seminal receptacle present. Mehlis’ gland and Laurer’s canal obscured by eggs. Uterus reaches from about mid-way between ventral sucker and ovary to posterior part of post-testicular region; distal extremity passes ventrally to cirrus-sac. Eggs numerous, small. Metraterm not differentiated, terminal part of uterus passes into anterior part of terminal organ through sphincter. Terminal organ lies alongside or ventral to cirrus-sac, of similar length to ejaculatory duct, lined with large spines which may appear to extend into base of genital atrium. Vitellarium follicular; follicles unite to form lobulate strings, in lateral fields reaching from about mid-way between ventral sucker and ovary to close to posterior extremity.

Excretory pore terminal. Excretory vesicle obscured by eggs.

***Opisthomonorcheides ovacutus*** **(Mamaev,** **1970** **) Machida,** **2011**

Syn. *Longimonorchis ovacutus* Mamaev, 1970^6^

*Host*: *Parastromateus niger* (Bloch) (Carangidae).

*Site in host*: Intestine.

*Localities*: Off Bali (Bali Fish Market) (29.vii.2013); Tegal, Java (6°52′47″S, 109°7′32″E; from frozen host); off Penang, Malaysia (5°17′7″N, 100°27′22″E, Coll: L. Tak Seng).

*Voucher specimens*: Off Indonesia: NBC MZBTr 232-237, ZMB E.7605-7610, BMNH 2016.10.7.1; off Malaysia: BMNH 1979.9.12.40-42.

*Representative DNA sequences*: ITS2 rDNA, two identical replicates (one submitted to GenBank KX839157).

*Type-host and locality*: *Parastromateus niger* (Bloch) (= *Formio niger*) (Carangidae), Gulf of Tonkin.

*References*: 1. Mamaev (1970); 2. Present study.

*Descriptions*: 1, 2.

*Host*: Carangidae: *Parastromateus niger* (Bloch) (1, 2).

*Distribution*: Gulf of Tonkin (1); off Bali (2); Malacca Strait off Penang, Malaysia (2); Java Sea off Tegal, Java (2).

### Description (Figs. 3–4)

[Based on 7 specimens from off Bali. Measurements in Table 1.] Body fairly large, narrow, elongate, distinctly wider at testicular level. Body spines tiny, in annular rows reaching almost to ovary. Oral sucker oval, subterminal, small, may be withdrawn into forebody to various depths. Ventral sucker rounded, larger than oral sucker. Forebody relatively long. Prepharynx long, narrow. Pharynx squat-pyriform, small. Oesophagus short, wide. Intestinal bifurcation in posterior forebody. Caeca wide anteriorly, terminate blindly close to posterior extremity, sometimes obscured by eggs.

Testis single, oval, close to posterior end of body. Cirrus-sac claviform, reaches to about mid-point between ventral sucker and ovary. Internal seminal vesicle oval, in posterior third of cirrus-sac. Pars prostatica elongate, narrow, with distinct gland-cell ducts arcing around it. Ejaculatory duct wide, narrows gradually anteriorly, lined with long spines. Genital atrium distinct, narrow, just reaches into hindbody or only reaches dorsally to ventral sucker. Genital pore median, in posterior forebody.

Ovary clearly tripartite, although overall outline may be weakly tri-lobed or almost circular, in posterior half of hindbody, pre-testicular, close to testis. Uterine seminal receptacle present. Mehlis’ gland obscured by eggs. Laurer’s canal opens dorsally to ovary. Uterus reaches from about mid-way between ventral sucker and ovary to posterior part of post-testicular region; distal extremity passes ventrally to cirrus-sac. Eggs numerous, small. Metraterm not differentiated, terminal part of uterus passes into anterior part of terminal organ. Terminal organ lies alongside or ventral to cirrus-sac, of similar length to ejaculatory duct, lined with large spines which may extend into base of genital atrium. Vitellarium follicular; follicles unite to form lobulate strings in lateral fields, reaching from about mid-way between ventral sucker and ovary to close to posterior extremity.

Excretory pore terminal. Excretory vesicle obscured by eggs.

***Opisthomonorcheides decapteri*** **Parukhin,** **1966** 7

*Host*: *Atule mate* (Cuvier) (Carangidae).

*Site in host*: Digestive tract.

*Localities*: Off Nouméa (Nouméa Fish Market), New Caledonia (05.xii.2008; Coll: J-L. Justine); Jakarta Bay, Indonesia (6°13′6″S, 106°48′8″E; Coll: S. Theisen: from frozen host).

*Voucher specimens*: Indonesia ZMB E.7611; New Caledonia MNHN JNC2814A, 3371C; BMNH 2016.10.7.3.

*Type-host and locality*: *Decapterus* sp. (first host named, and source of scientific name) (Carangidae), Gulf of Tonkin.

*References*: 1. Parukhin (1966); 2. Parukhin (1976); 3. Parukhin (1989); 4. Shen (1990); 5. Dutta & Manna (1998); 6. Hussein et al. (2011); 7. Machida (2011); 8. Present study.

*Descriptions*: 1, 4, 5, 7, 8.

*Definitive hosts*: Carangidae: *Decapterus* sp. (1, 2), *Alepes djedaba* (Forsskål) (= *Caranx djedaba*) (2), *Atule mate* (Cuvier) (= *Selar mate*, *Caranx mate*) (1, 2, 4, 8), *Carangoides armatus* (Rüppell) (= *Caranx armatus*) (2), *Carangoides equula* (Temminck & Schlegel) (= *Caranx equila*) (2), *Caranx* sp. (2), *Parastromateus niger* (Bloch) (5), *Pseudocaranx dentex* (Bloch & Schneider) (= *Caranx adsensionis*) (2), *Rastrelliger kanagurta* (Cuvier) (7), *Trachurus trachurus* (Linnaeus) (2); Stromateidae: *Pampus argenteus* (Euphrasen) (2); Macrouridae: *Coryphaenoides striatura* Barnard (3); Lethrinidae: *Lethrinus nebulosus* (Forsskål) (6).

*Distribution*: Gulf of Tonkin (1); South China Sea (2); Arabian Sea off Oman (2); Mozambique Channel (2, 3); Gulf of Mannar (2); South China Sea off Hainan Island (4); Bay of Bengal, Digha coast, India (5); Red Sea off Abha (from Abha fish market), Saudi Arabia (6); off Palawan, Philippines (7); Coral Sea off New Caledonia and Java Sea, Jakarta Bay, Indonesia (8).

### Description (Fig. 5)

[Based on 5 specimens from off New Caledonia. Measurements for 3 specimens from off New Caledonia presented in Table 1.] Body narrow, elongate, with sides nearly parallel but slightly wider between gonads. Body spines tiny, in annular rows reaching to level of cirrus-sac. Oral sucker oval, subterminal, small. Ventral sucker rounded, larger than oral sucker. Forebody relatively long. Prepharynx long, narrow. Pharynx squat-pyriform. Oesophagus short, wide. Intestinal bifurcation in posterior third of forebody. Caeca wide anteriorly, terminate close to posterior extremity, sometimes obscured by eggs.

Testis single, oval, fairly close to posterior extremity of body. Cirrus-sac claviform, reaches to about two thirds of distance from ventral sucker to ovary. Internal seminal vesicle oval, in posterior half of cirrus-sac. Pars prostatica elongate, narrow, with distinct gland-cell ducts arcing around it. Ejaculatory duct wide, lined with long spines. Genital atrium long, narrow or inflated, reaches well into hindbody. Genital pore median, in posterior forebody.

Ovary clearly tripartite, although overall outline may be weakly tri-lobed or almost circular, in mid-hindbody, pre-testicular, distant from testis. Uterine seminal receptacle observed posterior and anterior to ovary. Mehlis’ gland and Laurer’s canal obscured by eggs. Uterus reaches from about mid-way between ventral sucker and ovary to posterior part of post-testicular region; distal extremity passes ventrally to cirrus-sac. Eggs numerous, small. Metraterm not differentiated, terminal part of uterus passes into anterior part of terminal organ. Terminal organ lies alongside or ventral to cirrus-sac, of similar length to ejaculatory duct, lined with large spines. Vitellarium follicular, in lateral fields reaching from just posterior to mid-way between ventral sucker and ovary to anterior edge of testis.

Excretory pore terminal. Excretory vesicle obscured by eggs.

## Review of species

Our study has indicated that the 27 described members of *Opisthomonorcheides* can be divided conveniently into four groups based on the length of the genital atrium and the posterior extent of the vitellarium. The four groups are named after the most easily recognisable species. We emphasise that these characters have been selected for taxonomic rather than phylogenetic relevance and are used as an aid to identification only. The groups that can be distinguished are:

**Group A -** ***decapteri*** **group**: Long genital atrium reaching a long way into the hindbody; vitellarium restricted to pre-testicular region: *O. decapteri* (type-species) and *O. gibsoni* (Ahmad, 1991);

**Group B -** ***pampi*** **group**: Long genital atrium reaching well into hindbody; vitellarium extending distinctly beyond posterior edge of testis, often reaching close to posterior extremity: *O. pampi*, *O. chaetodoni* Ahmad, 1985, *O. jamilahmadi* Pozdnyakov, 1981, *O. madhavae* (Ahmad, 1984), *O. overstreeti* Ahmad, 1985, *O. simhai* Gupta & Puri, 1985 (syn. *O. engraulisi* Gupta & Singh, 1990) and *O. upeneusi* Ahmad, 1985;

**Group C -** ***karwarensis*** **group**: Short genital atrium reaching dorsally to ventral sucker or just into hindbody; vitellarium restricted to pre-testicular region: *O. karwarensis* Karyakarte & Yadav, 1976 (syns *O. delicatus* (Madhavi, 1977), *O. nigeri* Gupta & Puri, 1981) and *O. nahhasi* (Ahmad, 1985);

**Group D -** ***ovacutus*** **group**: Short genital atrium reaching dorsally to ventral sucker or just into hindbody; vitellarium extending distinctly beyond posterior margin of testis, often reaching close to posterior extremity: *O. ovacutus*, *O. aspinosus* Khan & Karyakarte, 1985, *O. guptai* (Ahmad, 1985), *O. indicus* Karyakarte & Yadav, 1976, *O. malabaricusi* Gupta & Puri, 1985, *O. manteri* (Ahmad, 1985), *Retractomonorchis overstreeti* Ahmad, 1985, *O. pellonai* Gupta & Puri, 1985, *O. shindei* Gupta & Puri, 1985, *O. stromatense* Ahmad & Gupta, 1985, *O. yamagutii* Gupta & Singh, 1990 and *O. yamagutinsis* Lokhande, 1993.

Unrecognisable: *Tritesticulum biovarium* Bilqees, 1971

**Group A:** ***decapteri*** **group**

The type-species, *O. decapteri,* is now fairly well known, based on the original description, those of Shen (1990) and Machida (2011) and that given here.

***Opisthomonorcheides decapteri*** **Parukhin,** **1966**

See above for summary of reports.

***Opisthomonorcheides gibsoni*** **(Ahmad,** **1991** **) Madhavi,** **2011**

Syn. *Retractomonorchis gibsoni* Ahmad, 1991^8^

*Type-host and locality*: *Cephalopholis sonnerati* (Valenciennes) (Serranidae), off Panjim, Goa, India.

*References*: 1. Ahmad (1991); 2. Al-Daraji et al. (2002).

*Hosts*: Serranidae: *Cephalopholis sonnerati* (1), *Epinephelus coioides* (Hamilton) (2).

*Distribution*: Arabian Sea off Panjim, Goa, India (1); Persian Gulf off Khawr Abd Allah, Iraq (2).

Remarks

This species differs from *O. decapteri* in the course of the metraterm, which is described and illustrated as entering the base of the rather small terminal organ. It was reported as *Retractomonorchis gibsoni* from *Epinephelus tauvina* (Forsskål) off Khaw Abd Allah, Iraq by Al-Daraji et al. (2002). Mhaisen et al. (2013) noted that *E. tauvina* is not found in the Arabian Gulf and that this is probably a misidentification of *E. coioides* (Hamilton); their interpretation is accepted here.

**Group B:** ***pampi*** **group**

***Opisthomonorcheides pampi*** **(Wang,** **1982** **) Liu, Peng, Gao, Fu, Wu, Lu, Gao & Xiao, 2010**

Remarks

The list of reports of this species is given above. We have interpreted some of the new specimens from off Bali as belonging to this species. The caeca were originally described as ‘united’ at the posterior extremity by Wang (1982). However, the terminations of the caeca are difficult to detect amongst the eggs in the new specimens, although, in a few cases, it is apparent that they end blindly. As this feature is difficult to detect in our specimens, we do not think this is a reliable feature to distinguish these forms. The illustration in the original description indicates that the uterus enters the terminal organ from the side.

***Opisthomonorcheides chaetodoni*** **Ahmad, 1985** 9

*Type-host and locality*: *Chaetodon vagabundus* Linnaeus (= *Chaetodon pictus*) (Chaetodontidae), Arabian Sea off Mumbai coast.

*Reference*: Ahmad (1985b).

Remarks

The ovary of this species is reported as being bilobed and the prepharynx and oesophagus as being of similar length. The metraterm is described and illustrated as entering the base of the terminal organ. These characteristics serve to distinguish this species from *O. pampi*.

***Opisthomonorcheides jamilahmadi*** **Pozdnyakov,** **1981** 10

Syns *Opisthomonorcheides indicus* Ahmad, 1977 *nec* Karyakarte & Yadav, 1976; *Opisthomonorcheides puriensis* Ahmad, 1985; *Opisthomonorchis puriensis* Ahmad & Gupta, 1985; *Opisthomonorcheides apogonichthysi* Gupta & Puri, 1985

*Type-host and locality*: *Apogon ellioti* Day (= *Apogonichthys ellioti*) (Apogonidae), Bay of Bengal off Orissa State, Puri, Bay of Bengal, India.

*Reference*: Ahmad (1977).

Remarks

This species was pre-occupied and has been renamed four times (Pozdnyakov, 1981; Ahmad, 1985b; Ahmad & Gupta, 1985; Gupta & Puri, 1985). The name *O. puriensis* was erected by both Ahmad (1985b) and Ahmad & Gupta (1985). The point of entry of the uterus into the terminal organ is not described, but the illustration suggests that it enters the base.

***Opisthomonorcheides madhavae*** **(Ahmad,** **1984** **) Madhavi,** **2011**

Syn. *Retractomonorchis madhavae* Ahmad, 1984^11^

*Type-host and locality*: *Cephalopholis sonnerati* (Valenciennes) (Serranidae), Arabian Sea off Mumbai coast.

*Reference*: Ahmad (1984).

Remarks

This is a distinctive species, with the testis close to the posterior extremity. The ovary is oval, the metraterm is described and illustrated as entering the base of the terminal organ and the oesophagus is much longer than the prepharynx.

***Opisthomonorcheides overstreeti*** **Ahmad, 1985** 12

*Type-host and locality*: *Pampus chinensis* (Euphrasen) (= *Chondroplitis chinensis*) (Stromateidae), Arabian Sea off the Mumbai coast.

*Reference*: Ahmad (1985b).

Remarks

This species is distinctive in having an equatorial ventral sucker and an oesophagus much longer than the prepharynx. The metraterm is described and illustrated as entering the base of the terminal organ. *Opisthomonorcheides overstreeti* (Ahmad, 1985) Madhavi 2011 is herein considered to be a junior homonym (see below).

***Opisthomonorcheides simhai*** **Gupta & Puri,** **1985** 13

Syn. *Opisthomonorcheides engraulisi* Gupta & Singh, 1990^14^

*Type-host and locality*: *Thryssa mystax* (Schneider) (= *Engraulis mystax*) (Engraulidae), Bay of Bengal off Puri, Orissa, India.

*References*: 1. Gupta & Puri (1985); 2. Gupta & Singh (1990).

*Descriptions*: 1, 2.

*Host*: *Thryssa mystax* (1, 2).

*Distribution*: Bay of Bengal off Puri, Orissa, India (1); Arabian Sea off Quilon, Kerala, India (2).

Remarks

This species was originally described with a bipartite seminal vesicle and the metraterm is described and illustrated as entering the base of the terminal organ (Gupta & Puri, 1985). Similarly, the seminal vesicle is described as bipartite and the uterus is described as entering the distal end of the terminal organ in the description of *O. engraulisi* (see Gupta & Singh, 1990). We considered *O. engraulisi* a junior synonym of *O. simhai* as it described from the same host, in the northern waters of the Indian Ocean, and we can detect no significant differentiating features.

***Opisthomonorcheides upeneusi*** **Ahmad, 1985** 15

*Type-host and locality*: *Upeneus sulphureus* Cuvier (Mullidae), Arabian Sea off the Mumbai coast.

*Reference*: Ahmad (1985b).

Remarks

This species is distinctive in possessing a vitellarium that is interrupted at the level of the ovary, a testis that is at the posterior extremity, a long oesophagus and a short prepharynx. The ovary is described as spherical and the metraterm is described and illustrated as entering the base of the terminal organ.

**Group C:** ***karwarensis*** **group**

***Opisthomonorcheides karwarensis*** **Karyakarte & Yadav, 1976** 16

Syns *Opisthomonorcheides delicatus* (Madhavi, 1977) Madhavi, 2011; *Retractomonorchis delicatus* Madhavi, 1977^17^; *Opisthomonorcheides nigeri* Gupta & Puri, 1981^18^

*Type-host and locality*: *Parastromateus niger* (Bloch), off Karwar, Arabian Sea, India.

*References*: 1. Karyakarte & Yadav (1976b); 2. Madhavi (1977); 3. Gupta & Puri (1981); 4. Hafeezullah (1984).

*Definitive host*s: Carangidae: *Parastromateus niger* (Bloch) (= *Stromateus niger*, *Formio niger*) (1, 3, 4); Stromateidae: *Pampus chinensis* (Euphrasen) (2), *P. argenteus* (Euphrasen) (2).

*Descriptions*. 1, 2, 3, 4.

*Distribution*: Arabian Sea off Karwar (1); Bay of Bengal off Waltair Coast, India (2); Arabian Sea off Kozhikode, Kerala, India (3); Bay of Bengal off Gopalpur (4); Arabian Sea off Mangalore (4).

Remarks

According to Hafeezullah (1984), *Opisthomonorcheides indicus* Karyakarte & Yadav, 1976, *O. indicus* Ahmad, 1977, *O. indicus* Gupta & Gupta, 1978, *R. delicatus* Madhavi, 1977 and ‘*Opisthomonorcheides indicus* Gupta & Puri, 1981 (lapsus for *nigeri*)’ are synonyms of *O. karwarensis*. He reckoned that only two *Opisthomonorcheides* species are valid, *O. karwarensis* and *O. decapterus.* It appears that this species is valid, being the oldest species with a pre-testicular vitellarium and a short genital atrium. The terminal organ is not described or illustrated in the original description.

We see no reason to disagree with the opinion of Hafeezullah (1984) who considered *O. delicatus* a synonym of *O. karwarensis* (under its original name *Retractomonorchis delicatus*). The uterus is described as entering the distal end of the terminal organ, which, judging from the illustration, likely means the base (Madhavi, 1977).

Similarly, we agree that *O. nigeri* is a synonym of *O. karwarensis*. Although Hafeezullah (1984) listed ‘*O. indicus* Gupta & Puri, 1981’ as a synonym of *O. karwarensis*, the text makes it clear that he is referring to *O. nigeri*. The terminal organ was not described or illustrated by Gupta & Puri (1981).

***Opisthomonorcheides nahhasi*** **(Ahmad, 1985) Madhavi, 2011**

Syn. *Retractomonorchis nahhasi* Ahmad, 1985^19^

*Type-host and locality*: *Epinephelus undulosus* (Quoy & Gaimard) (Serranidae), Arabian Sea off Mumbai, Maharashtra State, India.

*Reference*: Ahmad (1985a).

Remarks

This species is unusual in the possession of an entire ovary and, more especially, in the vitellarium reaching to the level of the ventral sucker. The uterus is described and illustrated as entering the base of the terminal organ.

**Group D:** ***ovacutus*** **group**

***Opisthomonorcheides ovacutus*** **(Mamaev, 1970) Machida, 2011**

Remark

The list of reports of this species is given above. The uterus is described and illustrated as entering the anterior part of the side of the terminal organ.

***Opisthomonorcheides aspinosus*** **Khan & Karyakarte, 1985** 20

*Type-host and locality*: *Pampus argenteus* (Euphrasen) (= *Stromateus argenteus*) (Stromateidae), Arabian Sea off Ratnagiri, India.

*Reference*: Khan & Karyakarte (1985).

Remarks

This species was reported to have a vitellarium that reaches the ‘posterior tip of the testis’, thus differing from *O. ovacutus*. The terminal organ was not described.

***Opisthomonorcheides guptai*** **(Ahmad, 1985) Madhavi, 2011**

Syn. *Retractomonorchis guptai* Ahmad, 1985^21^

*Type-host and locality*: *Drepane punctata* (Linnaeus) (Drepaneidae), Arabian Sea off the Bombay coast.

*Reference*: Ahmad (1985a).

Remarks

The status of this species remains unclear. It is reported to possess an unlobed ovary and a vitellarium that reaches to mid-testicular level. The uterus is described as entering the base of the terminal organ.

***Opisthomonorcheides indicus*** **Karyakarte & Yadav, 1976** 22

*Type-host and locality*: *Parastromateus niger* (Bloch) (Carangidae), off Ratnagiri and Karwar, Arabian Sea.

*References*: 1. Karyakarte & Yadav (1976a); 2. Shen (1990); 3. Shen & Tong (1990).

*Descriptions*: 1, 2.

*Definitive host*: *Parastromateus niger* (Bloch) (= *Stromateus niger*, *Formio niger*) (1, 2, 3).

*Distribution*: Arabian Sea off Ratnagiri and Karwar (1); South China Sea off Hainan Island (2); South China Sea, Daya Bay (3).

Remarks

This species has a vitellarium that reaches to the posterior margin of the testis, thus apparently differing from *O. ovacutus*. It is not to be confused with *O. indicus* Gupta & Gupta, 1978 (which is now known as *O. malabaricusi* Gupta & Puri, 1985) or *O. indicus* Ahmad, 1977 (now known as *O. jamilahmadi* Pozdnyakov, 1981). The terminal organ was not described.

***Opisthomonorcheides malabaricusi*** **Gupta & Puri, 1985** 23

Syns *Opisthomonorcheides indicus* Gupta & Gupta, 1978 *nec* Karyakarte & Yadav, 1976; *Retractomonorchis indicus* (Gupta & Gupta, 1978) Ahmad, 1984

*Type-host and locality*: *Carangoides malabaricus* (Bloch & Schneider) (= *Caranx malabaricus*) (Carangidae), Arabian Sea off Quilon, Kerala, India.

*Reference*: Gupta & Gupta (1978).

Remarks

For this species, which was renamed by Gupta & Puri (1985), the posterior vitelline extent is described as ‘either posterior 1st part or, hind end or some distance back of testis’. The uterus is described as ‘opening into terminal organ near its posterior side’.

***Opisthomonorcheides manteri*** **(Ahmad, 1985) Madhavi, 2011**

Syn. *Retractomonorchis manteri* Ahmad, 1985^24^

*Type-host and locality*: *Caranx sexfasciatus* Quoy & Gaimard (Carangidae), Arabian Sea off Mumbai, Maharashtra, India.

*Reference*: Ahmad (1985a).

Remarks

This species has a vitellarium described as ‘extending from a little posterior to posterior margin of acetabulum to hind end of testis’. The vitelline extent, therefore, does not coincide with that of *O. ovacutus*. The uterus is described as entering the base of the terminal organ.

***Opisthomonorcheides overstreeti*** **(Ahmad, 1985) Madhavi, 2011**, ***nec O. overstreeti*** **Ahmad, 1985**

Syn. *Retractomonorchis overstreeti* Ahmad, 1985^25^

*Type-host and locality*: *Scolopsis vosmeri* (Bloch) (Nemipteridae), Arabian Sea off Mumbai, Maharashtra, India.

*Reference*: Ahmad (1985a).

Remarks

Madhavi (2011) used the combination *O. overstreeti*, while fully aware that it is a homonym of *O. overstreeti* Ahmad 1985. The two names in question were erected in the same volume and part of the journal, but *Retractomonorchis overstreeti* was introduced on p. 19 and *Opisthomonorcheides overstreeti* on p. 250. According to the ICZN Article 24 (and see comment on http://iczn.org/content/there-such-thing-%E2%80%9Cpage-priority%E2%80%9D), priority relies on the actions of the first reviser, not page priority. As this name is a subsequent combination, the other species (*O*. *overstreeti* Ahmad, 1985) is here given priority (we consider the present study to be the first revision). The ovary is described as entire, and the worm is relatively squat, thus differing from *O. ovacutus*. The uterus is described as entering the base of the terminal organ.

***Opisthomonorcheides pellonai*** **Gupta & Puri, 1985** 26

*Type-host and locality*: *Ilisha elongata* (Bennett) (= *Pellona elongata*) (Pristigasteridae), Bay of Bengal off Puri, Orissa, India.

*Reference*: Gupta & Puri (1985).

Remark

The uterus is described as entering at the ‘distal end’ of the terminal organ. This is not clear in the illustration of Gupta & Puri (1985).

***Opisthomonorcheides shindei*** **Gupta & Puri, 1985** 27

*Type-host and locality*: *Apogon ellioti* Day (= *Apogonichthys ellioti*) (Apogonidae), Bay of Bengal off Puri, Orissa, India.

*Reference*: Gupta & Puri (1985).

Remarks

In the original description, the ovary is defined as being unlobed, but it is indented in the illustration. The uterus is described as entering the ‘distal’ end of the terminal organ, meaning the base, judging from the illustration.

***Opisthomonorcheides stromatensis*** **Ahmad & Gupta, 1985** 28 **emend.**

*Type-host and locality*: *Pampus argenteus* (Euphrasen) (= *Stromateus cinereus*) (Stromateidae), Bay of Bengal off Puri, Orissa, India.

*Other host*: *Parastromateus niger* (Bloch) (= *Stromateus niger*) (Carangidae).

*Reference*: Ahmad & Gupta (1985).

Remark

The uterus is described as entering the base of the terminal organ.

***Opisthomonorcheides yamagutii*** **Gupta & Singh, 1990** 29

*Type-host and locality*: *Psettodes erumei* (Bloch & Schneider) (Psettodidae), Bay of Bengal off Puri, Orissa, India.

*Reference*: Gupta & Singh (1990).

Remarks

The ovary is described as ‘unlobed’ and drawing of this species shows it to be relatively squat. The uterus is described as entering the ‘distal’ end of the terminal organ.

***Opisthomonorcheides yamagutinsis*** **Lokhande, 1993** 30

*Type-host and locality*: *Pampus argenteus* (Euphrasen) (Stromateidae), Arabian Sea off Alibag, Maharashtra, India.

*Reference*: Lokhande (1993).

Remarks

This species is drawn as rather squat and the testis is relatively tiny thus differing from *O. ovacutus*. The terminal organ is not described or illustrated.

**Unrecognisable**

***Tritesticulum biovarium*** **Bilqees, 1971** 31

*Type-host and locality*: *Stromateus* sp. (Stromateidae), Arabian Sea off Karachi coast, Pakistan.

*Reference*: Bilqees (1971).

Remark

Madhavi (2008) considered *Tritesticulum* a synonym of *Opisthomonorcheides,* stating ‘but its description is inadequate and probably erroneous’. It is unrecognisable.

**Molecular results**

ITS2 rDNA sequence data were generated for two individuals each of *O. pampi* and *O. ovacutus*. Sequences of *O. pampi* were 461 bp long, comprising 123 bp of flanking 5.8S, 289 bp of ITS2 and 49 bp of flanking 28S rDNA. The sequences of *O. ovacutus* were 470 bp long, comprising 123 bp of flanking 5.8S, 298 bp of ITS2 and 49 bp of flanking 28S rDNA. Replicate sequences of *O*. *pampi* and *O*. *ovacutus* were identical, but the two species differed at 12 positions in the ITS2 region (gaps excluded). Currently there are no sequence data for other species of *Opisthomonorcheides* in GenBank for comparison.

## Discussion

### Overview

In our view, the recognition of species in this genus is unusually problematic. It is unlikely that all nominal species are valid given the poor standard of many descriptions and the lack of critical comparison of the species at the time of description. However, we refrain from proposing sweeping synonymies because we think the issues are further confused by morphological uncertainty (especially with respect to the nature of the terminal genitalia) and host specificity (especially with respect to the infection of species of *Pampus* and *Parastromateus* but also with respect to the significance of infection of other groups of fishes). In combination, we conclude that these complexities make it difficult to ‘draw a line’ with respect to what might reasonably be considered distinct species. In addition, we note that we are unable to predict with any reliability whether there might be a rich complex of species of *Opisthomonorcheides* within individual fish species as has been shown for some other combinations of fish and trematodes (e.g. Miller & Cribb, 2007). Overall, we conclude that the true richness of this genus can only be resolved by new careful work which incorporates molecular data. However, we consider the most pressing issue to be that of whether the unrelated species of *Pampus* and *Parastromateus* are genuinely both infected by species of *Opisthomonorcheides*. With respect to the 27 described members of *Opisthomonorcheides* discussed above, we consider 11 of them (*O. decapteri*, *O. gibsoni*, *O. karwarensis*, *O. madhavae*, O*. manteri*, *O. nahhasi*, *O. ovacutus*, *O. overstreeti* Ahmad, 1985, *O. pampi*, *O. simhai* and *O. upeneusi*) clearly distinguishable from each other, along with 12 other less easily recognisable species; these are divided into groups with two species within group A, seven within group B, two within group C and 12 within group D.

### Terminal organ

In the key to the Monorchiidae of Madhavi (2008), the terminal organ of *Opisthomonorcheides* is diagnosed as having the uterus enter distally. However, here we have shown that what we interpret as specimens of the type-species, *O. decapteri*, as well as of *O. ovacutus* and *O. pampi*, clearly have the uterus entering the terminal organ laterally, well anterior to its posterior end. In Madhavi’s review of monorchiid genera, the conformation of the terminal organ with respect to the entry of the uterus is reported as non-varying for species of individual genera. In this context it is striking that, for 16 of the species of *Opisthomonorcheides* revised above, the entry of uterus into the terminal organ is described or figured as being terminal. Indeed, it is reported or illustrated as entering laterally only in the descriptions given here and for the original descriptions of *O. pampi* and *O. ovacutus,* plus the description of *O. decapteri* by Machida (2011). A number of the figures of terminal organs with a posterior entry of the uterus look convincing (e.g. Ahmad, 1984, 1991). We can detect no pattern of association between the reported conformation of the terminal genitalia and other aspects of the morphology or host distribution, and can only suggest that the subject needs further active consideration. This distinction may be a basis for a future subdivision of the genus.

### Host distribution

A critical component of the recognition of the validity of species of *Opisthomonorcheides* appears to lie in the nature of their host-specificity. In the host-parasite list (Table 2) 57 host records have been reported in the literature, including those in the present study. The hosts recorded encompass 14 families, the Carangidae 31 records (54%), Stromateidae 9 (16%), Serranidae 4 (7%), Apogonidae 2 (3.5%), Engraulidae 2 (3.5%), Chaetodontidae 1 (1.75%), Drepaneidae 1 (1.75%), Lethrinidae 1 (1.75%), Macrouridae 1 (1.75%), Mullidae 1 (1.75%), Nemipteridae 1 (1.75%), Percichthyidae 1 (1.75%), Pristigasteridae 1 (1.75%) and Psettodidae 1 (1.75%). Species of four fish orders have been recorded to be infected with species of this genus, the Perciformes 51 (89%), Clupeiformes 3 (5.3%), Gadiformes 1 (1.75%) and Pleuronectiformes 1 (1.75%).

**Table 2:** Host-parasite list of *Opisthomonorcheides* spp.

Host order	Host family	Host species	Parasite species	Records
Gadiformes	Macrouridae	*Coryphaenoides striatura* Barnard	*O. decapteri* Parukhin, 1966	1
Clupeiformes	Engraulidae	*Thryssa mystax* (Schneider)	*O. simhai* Gupta & Puri, 1985 (also as *O. engraulisi* Gupta & Singh, 1990)	2
	Pristigasteridae	*Ilisha elongata* (Bennett)	*O. pellonai* Gupta & Puri, 1985	1
Perciformes	Apogonidae	*Apogon ellioti* Day	*O. jamilahmadi* Pozdnyakov, 1981	1
			*O. shindei* Gupta & Puri, 1985	1
	Carangidae	*Alepes djedaba* (Forsskål)	*O. decapteri* Parukhin, 1966	1
		*Atule mate* (Cuvier)	*O. decapteri* Parukhin, 1966	5
		*Carangoides armatus* (Rüppell)	*O. decapteri* Parukhin, 1966	1
		*Carangoides equula* (Temminck & Schlegel)	*O. decapteri* Parukhin, 1966	1
		*Carangoides malabaricus* (Bloch & Schneider)	*O. malabaricusi* Gupta & Puri, 1985	1
		*Caranx sexfasciatus* Quoy & Gaimard	*O. manteri* (Ahmad, 1985)	1
		*Caranx* sp.	*O. decapteri* Parukhin, 1966	1
		*Decapterus* sp.	*O. decapteri* Parukhin, 1966	2
		*Parastromateus niger* (Bloch)	*O. decapteri* Parukhin, 1966	1
			*O. indicus* Karyakarte & Yadav, 1976	3
			*O. karwarensis* (also as *O. nigeri* Gupta & Puri, 1981)	4
			*O. ovacutus* (Mamaev, 1970)	4
			*O. pampi* (Wang, 1982)	2
			*O. stromatensis* Ahmad & Gupta, 1985	1
		*Pseudocaranx dentex* (Bloch & Schneider)	*O. decapteri* Parukhin, 1966	1
		*Rastrelliger kanagurta* (Cuvier)	*O. decapteri* Parukhin, 1966	1
		*Trachurus trachurus* (Linnaeus)	*O. decapteri* Parukhin, 1966	1
	Chaetodontidae	*Chaetodon vagabundus* Linnaeus	*O. chaetodoni* Ahmad, 1985	1
	Drepaneidae	*Drepane punctata* (Linnaeus)	*O. guptai* (Ahmad, 1985)	1
	Lethrinidae	*Lethrinus nebulosus* (Forsskål)	*O. decapteri* Parukhin, 1966	1
	Mullidae	*Upeneus sulphureus* Cuvier	*O. upeneusi* Ahmad, 1985	1
	Nemipteridae	*Scolopsis vosmeri* (Bloch)	*O. overstreeti* (Ahmad, 1985) *nec* Ahmad, 1985	1
	Percichthyidae	*Lateolabrax japonicus* (Cuvier)	*O. pampi* (Wang, 1982)	1
	Serranidae	*Cephalopholis sonnerati* (Valenciennes)	*O. gibsoni* (Ahmad, 1991)	1
			*O. madhavae* (Ahmad, 1984)	1
		*Epinephelus coioides* (Hamilton)	*O. gibsoni* (Ahmad, 1991)	1
		*Epinephelus undulosus* (Quoy & Gaimard)	*O. nahhasi* (Ahmad, 1985)	1
	Stromateidae	*Pampus argenteus* (Euphrasen)	*O. aspinosus* Khan & Karyakarte, 1985	1
			*O. decapteri* Parukhin, 1966	1
			*O. karwarensis* (as *O. delicatus* (Madhavi, 1977))	1
			*O. pampi* (Wang, 1982)	1
			*O. stromatensis* Ahmad & Gupta, 1985	1
			*O. yamagutinsis* Lokhande, 1993	1
		*Pampus chinensis* (Euphrasen)	*O. delicatus* (Madhavi, 1977)	1
			*O. overstreeti* Ahmad, 1985	1
		*Stromateus* sp.	*Tritesticulum biovarium* Bilqees, 1971	1
Pleuronectiformes	Psettodidae	*Psettodes erumei* (Bloch & Schneider)	*O. yamagutii* Gupta & Singh, 1990	1

The most interesting aspect of the host distribution of species of *Opisthomonorcheides* is the reported concentration of species in the black pomfret (*Parastromateus niger*), which has six recorded species and the silver pomfret (*Pampus argenteus*) with six species. In addition, the Chinese silver pomfret (*P. chinensis*) has two reported species. Despite the remarkable morphological similarity of *Parastromateus* and *Pampus*, these two genera are considered to belong to different families, the Carangidae and Stromateidae respectively; indeed, according to WoRMS (Bailly, 2008), the two families belong to separate suborders of the Perciformes. Two *Opisthomonorcheides* species (*O. decapteri* and *O. pampi*) are reported as being shared by these families. This distribution may be a striking case of morphological (and presumably dietary) convergence between the two groups of fishes, leading to the sharing of parasites. Alternatively, there may have been a series of misidentifications of some of the hosts. If the latter explanation is to hold, then we would predict that it is the stromateid hosts that have been misidentified, given that numerous additional carangids have been reported as hosts.

The credibility of a number of the remaining host records (and thus *Opisthomonorcheides* species) is suspect. In our view, the evidence is clear that monorchiid species, in general, and species of *Opisthomonorcheides*, in particular, typically infect single or only closely related species, thus illustrating oioxenous or stenoxenous specificity. Accordingly, of all the species of *Opisthomonorcheides*, just two have been reported from multiple fish families. *Opisthomonorcheides pampi* has been reported from a stromateid, a carangid and a percichthyid. The sharing between a stromateid and a carangid is a special case, as discussed above. In our view, the record of *O. pampi* from a percichthyid is either not credible, accidental or not significant. We think that it is not plausible that a species should be shared by two such distantly related and ecologically different fishes but seemingly not by other, more closely related, sympatric hosts. The second species from different host families, *O. decapteri*, has been reported from multiple carangids, a stromateid, a macrourid and a lethrinid. In his discussion of the digenean parasites of macrourids, Bray (1995) cast doubt on the record from that family, pointing out that most other records are from pelagic carangids. Similarly, the record of *O. decapteri* from a lethrinid is based on a single highly macerated (as photographed) specimen with, apparently, two testes (Hussein et al., 2011). We contend that the single specimen from a distantly related and ecologically different fish is not plausible.

Beyond the dominant records from carangids and stromateids, several species have been reported exclusively from other families: Apogonidae (*O. jamilahmadi*, *O. shindei*); Chaetodontidae (*O. chaetodoni*); Engraulidae (*O. simhai* [syn. *O. engraulisi*]); Drepaneidae (*O. guptai*); Mullidae (*O. upeneusi*); Nemipteridae (*O. overstreeti* (Ahmad, 1985) *nec* Ahmad, 1985); Pristigasteridae (*O. pellonai*); Psettodidae (*O. yamagutii*); Serranidae (*O. decapteri*, *O. madhavae*, *O. nahhasi*).

Despite the dramatic taxonomic differences between many of these families and the Carangidae and Stromateidae, many of the records and species appear valid given that they have been reported on the basis of multiple specimens. In this category are the reports of *O. gibsoni*, *O. chaetodoni*, *O. upeneusi*, *O. madhavae*, *O. nahhasi*, *O. guptai* and *O. overstreeti* (Ahmad, 1985) Madhavi, 2011. In contrast, *O. engraulisi* and *O. simhai* were described on the basis of just two specimens each from engraulids. The discussion of *O. engraulisi* does not mention *O. simhai*. The two species are strikingly similar and we consider the former a synonym of the latter. There is a precedent for such a pattern of host-specificity (rich in one family, scattered in others) among members of the Monorchiidae. Although species of *Hurleytrematoides* Yamaguti, 1954 occur overwhelmingly in chaetodontid fishes (22 species), a handful of species are reported from the Acanthuridae, Pomacanthidae, Siganidae and Tetraodontidae (see McNamara & Cribb, 2011).

### Molecular data

It is our hope that the reporting of ITS2 rDNA sequences relating to *O. pampi* and *O. ovacutus* here will assist in the future recognition and delineation of species in this genus. The only other monorchiid genus for which there are sequence data available for multiple species is *Hurleytrematoides.* McNamara et al. (2014) successfully used ITS2 rDNA sequences (in parallel with mtCO1 sequences) to distinguish 16 species of *Hurleytrematoides* from chaetodontid and tetraodontid fishes in the Tropical Indo-west Pacific. In that study, interspecific differences in the ITS2 region ranged from 8 to 84 bp. Analysis of the new data indicates that ITS2 sequences of sympatric *O. pampi* and *O. ovacutus* differed by 12 bp, a level which is consistent with the interspecific difference seen between species of *Hurleytrematoides* and in other trematode groups (Diaz et al., 2015; Nolan et al., 2016; Yong et al., 2016). We note that McNamara et al. (2014) reported differences up to 9 bp in the ITS2 region between samples of the morphologically distinctive species *H. morandi* McNamara & Cribb, 2011 from off the Great Barrier Reef and Palau. This was interpreted as geographical intraspecific variation; we thus caution that future comparisons of species of *Opisthomonorcheides* should expect, or at least allow for, the possibility of similar variation.

## Footnotes

1 urn:lsid:zoobank.org:act:22FE37CC-5D9E-4C61-ACB2-275FAA83101D.

2 urn:lsid:zoobank.org:act:B76D2785-F356-46A3-9299-CEF0A359F3D5.

3 urn:lsid:zoobank.org:act:37E7FEFF-2256-4F11-957A-C3891289D697.

4 urn:lsid:zoobank.org:act:EC0947FE-208B-40CD-A187-6B971202F401.

5 urn:lsid:zoobank.org:act:CED8B7F8-D8BA-4066-BF93-F6CAE6681D10.

6 urn:lsid:zoobank.org:act:CF4C2576-6066-41BA-8D23-AE67196445DB.

7 urn:lsid:zoobank.org:act:31B5215B-BEC1-4343-9B56-D857896553A4.

8 urn:lsid:zoobank.org:act:F5FC8531-AA37-479F-9F7F-8101D107C537.

9 urn:lsid:zoobank.org:act:407D22CD-4179-4E8D-8D34-2E9A51EE6526.

10 urn:lsid:zoobank.org:act:8B46146D-44E1-4CBB-AE66-1C5D8D93CA78.

11 urn:lsid:zoobank.org:act:25CC6E4D-BF65-403E-9977-12F928407F50.

12 urn:lsid:zoobank.org:act:8D1A5D4B-90E3-4144-8DFC-167B186FF317.

13 urn:lsid:zoobank.org:act:FC9AE97D-BAB0-4635-A2BC-A06DD3D70F2A.

14 urn:lsid:zoobank.org:act:34E5935D-A023-45F1-9A4F-15A978B85946.

15 urn:lsid:zoobank.org:act:0CD0BC84-1179-4FB5-806A-1C97AD2524F7.

16 urn:lsid:zoobank.org:act:99D3CA25-F655-4ACF-A19A-81FA2E0E4992.

17 urn:lsid:zoobank.org:act:534FF540-BA44-4F4F-9318-5B571A0482AF.

18 urn:lsid:zoobank.org:act:DC55B113-E541-4B18-8097-F5745A5E2CEA.

19 urn:lsid:zoobank.org:act:62E01DD1-2277-40FC-9F8E-659277925F62.

20 urn:lsid:zoobank.org:act:937FCD30-BA92-4F57-9FEB-51AB79112A80.

21 urn:lsid:zoobank.org:act:1527292C-9F60-460D-BE98-11E96A24ED98.

22 urn:lsid:zoobank.org:act:859409F3-661E-41C6-A791-7B5B7B1FC3A7.

23 urn:lsid:zoobank.org:act:03AFED65-AED2-492F-8A59-F325095BCDCC.

24 urn:lsid:zoobank.org:act:0823883A-32ED-4390-963C-5B36564F66CA.

25 urn:lsid:zoobank.org:act:20A1AC28-C626-4F82-9978-D575BB5802F4.

26 urn:lsid:zoobank.org:act:83B6EE0B-D42E-4407-89E6-57735C92340F.

27 urn:lsid:zoobank.org:act:5503AAD7-5E48-4460-AE10-E5387F36FFF3.

28 urn:lsid:zoobank.org:act:95334C2A-50E4-4227-A4F6-5CBC64C25C33.

29 urn:lsid:zoobank.org:act:04EE1076-1BD3-4E11-9815-FCF7D4F9F3D7.

30 urn:lsid:zoobank.org:act:5EDA8EC3-86EE-4FC1-AAFF-0F7A60A382C7.

31 urn:lsid:zoobank.org:act:C2730D64-F493-4D27-A64D-46342BD04FD7.
